# TLX1 and NOTCH coregulate transcription in T cell acute lymphoblastic leukemia cells

**DOI:** 10.1186/1476-4598-9-181

**Published:** 2010-07-09

**Authors:** Irene Riz, Teresa S Hawley, Truong V Luu, Norman H Lee, Robert G Hawley

**Affiliations:** 1 Department of Anatomy and Regenerative Biology, The George Washington University Medical Center, Washington, DC, USA; 2 Flow Cytometry Core Facility, The George Washington University Medical Center, Washington, DC, USA; 3 Department of Pharmacology and Physiology, The George Washington University Medical Center, Washington, DC, USA

## Abstract

**Background:**

The homeobox gene *TLX1 *(for *T-cell leukemia homeobox 1*, previously known as *HOX11*) is inappropriately expressed in a major subgroup of T cell acute lymphoblastic leukemia (T-ALL) where it is strongly associated with activating *NOTCH1 *mutations. Despite the recognition that these genetic lesions cooperate in leukemogenesis, there have been no mechanistic studies addressing how *TLX1 *and *NOTCH1 *functionally interact to promote the leukemic phenotype.

**Results:**

Global gene expression profiling after downregulation of TLX1 and inhibition of the NOTCH pathway in ALL-SIL cells revealed that TLX1 synergistically regulated more than 60% of the NOTCH-responsive genes. Structure-function analysis demonstrated that TLX1 binding to Groucho-related TLE corepressors was necessary for maximal transcriptional regulation of the NOTCH-responsive genes tested, implicating TLX1 modulation of the NOTCH-TLE regulatory network. Comparison of the dataset to publicly available biological databases indicated that the TLX1/NOTCH-coregulated genes are frequently targeted by MYC. Gain- and loss-of-function experiments confirmed that MYC was an essential mediator of TLX1/NOTCH transcriptional output and growth promotion in ALL-SIL cells, with TLX1 contributing to the NOTCH-MYC regulatory axis by posttranscriptional enhancement of MYC protein levels. Functional classification of the TLX1/NOTCH-coregulated targets also showed enrichment for genes associated with other human cancers as well as those involved in developmental processes. In particular, we found that TLX1, NOTCH and MYC coregulate *CD1B *and *RAG1*, characteristic markers of early cortical thymocytes, and that concerted downregulation of the TLX1 and NOTCH pathways resulted in their irreversible repression.

**Conclusions:**

We found that TLX1 and NOTCH synergistically regulate transcription in T-ALL, at least in part via the sharing of a TLE corepressor and by augmenting expression of MYC. We conclude that the TLX1/NOTCH/MYC network is a central determinant promoting the growth and survival of TLX1^+ ^T-ALL cells. In addition, the TLX1/NOTCH/MYC transcriptional network coregulates genes involved in T cell development, such as CD1 and RAG family members, and therefore may prescribe the early cortical stage of differentiation arrest characteristic of the TLX1 subgroup of T-ALL.

## Background

Homeodomain-containing transcription factors play a major role in the establishment of metazoan body plans and organogenesis. They are also involved in the maintenance of tissue homeostasis, influencing the self-renewal and differentiation of stem cells and their progenitors. A number of experimental investigations have demonstrated that homeodomain transcription factors regulate multiple cellular functions including cell growth, proliferation, apoptosis, communication, adhesion and migration [1,2]. It is not surprising therefore that anomalous expression of homeobox genes can disrupt developmental programs and contribute to neoplasia [3,4].

*TLX1 *is an evolutionarily conserved member of the NKL (NK-Like or NK-Linked) subclass of Antennapedia homeobox genes. During normal development, *TLX1 *is required for the formation of the spleen and participates in certain neuronal cell fate decisions [5-7]. Although *TLX1 *is not normally expressed in the hematopoietic system, its inappropriate expression due to chromosomal translocations involving T cell receptor (TCR) genes is associated with about 30% of adult and approximately 8% of childhood T-cell acute lymphoblastic leukemia (T-ALL) cases [3,8]. T cell transforming activity of *TLX1 *has been confirmed experimentally in studies of murine bone marrow transplant recipients that received hematopoietic stem cells expressing a retrovirally-delivered *TLX1 *transgene [9,10]. However, a long latency of *TLX1*-induced tumorigenesis indicated the necessity for additional genetic abnormalities. In this regard, mutations activating *NOTCH1 *are observed in virtually all TLX1^+ ^T-ALL samples [11-13], arguing that the two factors frequently cooperate in the neoplastic conversion of T cell progenitors. NOTCH stimulates the PI3K-AKT-mTOR pathway and transcriptionally activates the NF-κB, MYC and HES1 transcription factors in T-ALL cells, but the critical target genes responsible for the NOTCH1-induced malignant phenotype remain to be fully defined [14-19].

The NOTCH receptor family plays an important role in T cell development by providing instructional and growth promoting signals [20,21]. Intrathymic T cell differentiation is associated with sequential changes in the expression of the CD1, CD3, CD4 and CD8 cell surface markers [22,23]. Early thymocyte precursors do not express CD3, CD4 or CD8. In-frame TCRβ rearrangement and the generation of a functional pre-TCR complex (TCRβ/pre-TCRα/CD3) at the cell surface allows continued thymocyte development via the process of β-selection. In humans, CD4 is transiently upregulated following β-selection and the immature single positive (ISP) CD4^+ ^cells rapidly give rise to CD4^+^CD8^+ ^double positive (DP) cells which undergo further maturation toward two distinct populations represented by CD4^+ ^or CD8^+ ^single positive (SP) phenotypes. NOTCH and/or pre-TCR signaling provide survival and trophic functions until the late DP stage when the cells become highly positive for CD3 and depend on TCR signaling [24,25]. Specifically, NOTCH signaling was shown to be obligatory for β-selection [26], and the direct transcriptional target of NOTCH, *MYC*, is a central integrator of NOTCH-mediated survival [27,28] and preTCR-mediated proliferative signals [29,30]. Surface expression of the CD1 family of genes increases until the late DP stage, after which their expression is extinguished and remains off in CD4^+ ^and CD8^+ ^SP cells [31,32]. TLX1^+ ^T-ALL samples exhibit a predominantly CD1^+^CD3^- ^surface phenotype with high levels of TCR recombination activating gene (*RAG1*) expression and TCRβ rearrangement on at least one allele; but they often lack cytoplasmic and surface TCRβ expression, suggesting that the onset of malignant transformation occurs before β-selection [32-36]. However TLX1^+ ^leukemic cells are characteristically CD4^+^CD8^+^, indicating that the oncogenic network allows the cells to bypass the β-selection checkpoint and arrest at the early cortical DP stage.

The direct transcriptional targets of NOTCH are tightly regulated such that they are repressed in the absence of a NOTCH activating signal [12,37]. In *Drosophila*, Groucho, a homolog of Transducin-like Enhancer-of-split (TLE) proteins, directly associates with the Suppressor of Hairless (the homolog of human RBP-Jκ) repressor complex to block transcription from NOTCH-responsive elements [38]. In mammals, SPEN/SHARP, a different RBP-Jκ-associated corepressor protein performs this function [39]. Instead, as was shown for the best characterized target of NOTCH, *HES1 *(reviewed in [40]), TLE proteins are involved in modulating NOTCH output by a mechanism that involves autorepression of HES1 via formation of a repressive HES1-TLE complex that recognizes an N-box sequence in the *HES1 *promoter [41]. Moreover, the levels of HES1-repressive activity have been shown to define the type of NOTCH response, e.g., whether it is repressed, oscillating or strongly activated [42]. It is noteworthy that transcriptional repression in response to NOTCH signaling occurs via an indirect mechanism through downstream effectors; among these, HES1-TLE repressor complexes play a central role, a well-studied example of which is in the context of NOTCH antineurogenic activity [43]. Thus, TLE corepressors contribute to two aspects of NOTCH signaling: establishing negative loops of regulation and mediating NOTCH repressor activity. TLE proteins are also emerging as focal points for crosstalk between signaling pathways [44,45]. Additionally, there is mounting evidence that the expression of TLE genes is altered in a growing list of human cancers [46], including hematologic malignancies [41,47].

We recently showed that TLX1 interacts with TLE1 via an Engrailed homology 1 (Eh1) motif (FXIXXIL, where X can be any amino acid) encompassing amino acids 19-26 [46,48], and proposed that TLX1 activates transcription at least in part by derepressing TLE-controlled genes [49]. Here we demonstrate that TLX1-TLE interaction contributes in a positive manner to NOTCH transcriptional programs in T-ALL. Functional classification of the overlapping targets of these cooperating genetic lesions showed enrichment for genes associated with other human cancers as well as those involved in developmental processes. We found that TLX1 augments the NOTCH-MYC regulatory axis by enhancing MYC protein levels and that this represents a major component of TLX1-mediated growth control in ALL-SIL cells. We further show that MYC coregulates a significant proportion of common TLX1/NOTCH targets, among them the *CD1B *and *RAG1 *genes characteristic of the early cortical phenotype exhibited by TLX1^+ ^T-ALLs [32-36]. Downregulation of TLX1 in concert with NOTCH blockade resulted in irreversible repression of these genes. Therefore, our data suggest that the TLX1/TLE/NOTCH/MYC network contributes to the pathogenesis of T-ALL by concomitantly promoting the differentiation arrest and expansion of cells at the CD1^+ ^early cortical DP stage of thymocyte development.

## Results

### TLX1 signature genes in T-ALL cells

**Figure 1:**
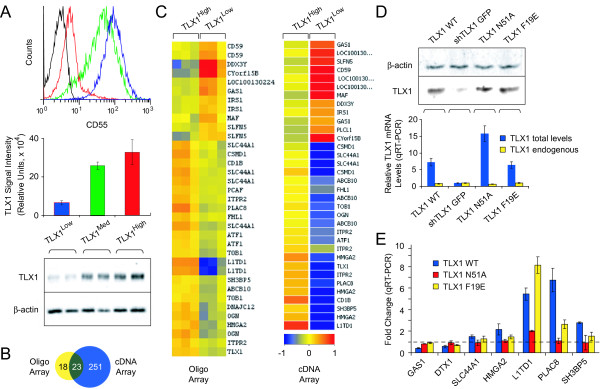
**Structure-function analysis of TLX1-mediated transcriptional regulation in ALL-SIL T-ALL cells**. (A) TLX1 protein levels inversely correlate with CD55 surface levels. Top, CD55 FACS analysis. Histogram colors indicate: blue, TLX1^Low^; green, TLX1^Med^; and red, TLX1^High^. Bottom, TLX1 Western blot. TLX1^High ^levels were 6.5× higher and TLX1^Med ^levels were 4.9× higher than TLX1^Low ^levels (*P *< 0.0001). The difference between the TLX1^High ^and TLX1^Med ^levels was statistically significant (*P *< 0.05). (B) Venn diagram comparing candidate TLX1 target genes derived from two different microarray technologies: Affymetrix data have been described [50]; cDNA array targets were selected based on correlation of expression levels with TLX1 protein levels (*r *> 0.9 or < -0.9; 1% FDR; *n *= 3-4). (C) Hierarchical clustering of genes identified in both profiling experiments. (D) TLX1 levels in knockdown and "rescued" ALL-SIL populations: top, Western blot; bottom qRT-PCR. The TLX1 knockdown populations expressing coding regions of wild-type or mutant forms of TLX1 are: TLX1 WT, wild-type; TLX1 N51A, DNA binding-deficient mutant; TLX1 F19E, TLE binding-deficient mutant; shTLX1 GFP, TLX1 knockdown plus GFP reporter. Total or endogenous TLX1 mRNA levels were determined using primers targeting coding or 3' noncoding regions of TLX1, respectively. The data is normalized to TLX1 knockdown expressing GFP alone (shTLX1 GFP). (E) TLX1 signature gene expression in the populations described in D. The data is normalized to TLX1 knockdown expressing GFP alone (*P *values are indicated in the text and Additional file 4; *n *= 2-3 biological replicates, each comprising 3 technical replicates).

In an effort to identify TLX1 target genes critical to the malignant phenotype in T-ALL, we recently performed gene expression profiling of a patient-derived TLX1^+ ^T-ALL cell line (ALL-SIL) where TLX1 was downregulated by a lentiviral TLX1 shRNA knockdown approach [50]. Despite selection for vector-encoded drug resistance, there was a certain degree of heterogeneity in TLX1 levels within the transduced cells [50]. We therefore sought to devise a strategy to obtain more homogeneous ALL-SIL populations, ideally having more defined levels of TLX1 expression. In that study, we observed that downregulation of TLX1 was associated with decreased levels of *CD1B *and increased levels of *CD55 *at the mRNA level and on the cell surface [50]. Based on those findings, we used fluorescence-activated cell sorting (FACS) to isolate the following ALL-SIL populations: cells with shRNA-mediated knockdown of TLX1 were sorted for a CD1b^Low^CD55^High ^surface phenotype (predicted "low" TLX1), and control vector-transduced cells were sorted into CD55^High ^(predicted intermediate or "medium" TLX1) and CD55^Low ^(predicted "high" TLX1) populations. As anticipated, TLX1 protein levels in the resulting populations exhibited an inverse correlation with surface expression of CD55 (*r *= -0.9) (Figure 1A). We performed expression profiling of the sorted TLX1^High^, TLX1^Med ^and TLX1^Low ^ALL-SIL cells to identify transcripts whose changes in expression levels correlated with TLX1 protein levels. To globally validate and extend our previous data, which was carried out using an Affymetrix oligonucleotide microarray [50], we chose a 39,936 element cDNA microarray for these experiments [51] (see ref. [52]) (Additional file 1). The datasets of candidate TLX1-responsive genes obtained using these two expression platforms overlapped significantly (*P *< 0.0001), yielding a robust consensus set of TLX1 signature genes (Figure 1B, C).

**Figure 2:**
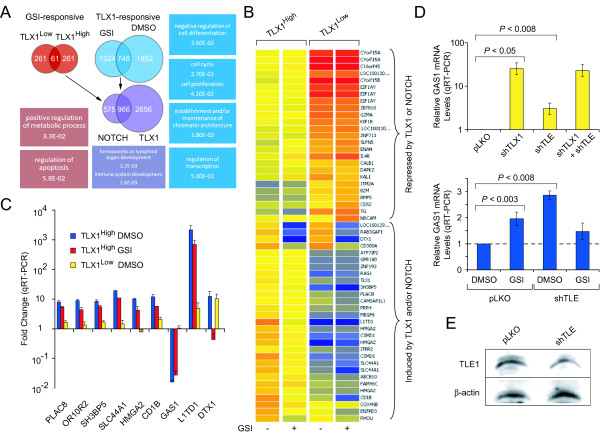
**TLX1 and NOTCH coregulate transcription**. (A) ALL-SIL cells were treated for 24 hours with 500 nM Compound E (GSI) and 0.05% DMSO as vehicle control and then harvested for microarray analysis. The Venn diagrams indicate a significant overlap between TLX1 targets and GSI-responsive genes (*P *< 0.0001; data for a 10% FDR is shown). GO terms uniquely associated with the overlapping genes in each pair-wise comparison are indicated (see text for details). (B) Hierarchical clustering of known genes showing > 2-fold change under TLX1^High ^versus TLX1^Low ^conditions and a FDR < 10%. (C) Confirmation by qRT-PCR of representative coregulated genes. Data is normalized to TLX1^Low ^GSI-treated samples, where both NOTCH and TLX1 are downregulated. The average of 3 experiments is shown (*P *values are indicated in the text where discussed and the *P *values for all comparisons are provided in Additional file 4; *n *= 3 biological replicates, each comprising 3 technical replicates). (D) TLX1 and NOTCH repress the *GAS1 *gene via a TLE corepressor-mediated mechanism. Top, ALL-SIL cells expressing the TX1 shRNA95 or pLKO control vector were transduced with lentiviral vectors expressing panTLE shRNAs. Bottom, ALL-SIL cells expressing the pLKO control vector were transduced with lentiviral vectors expressing panTLE shRNAs. GSI treatment was for 24 hours. (E) Western blot analysis showing 50% reduced TLE1 levels in ALL-SIL cells expressing panTLE shRNAs (*P *< 0.05).

To rule out off-target effects of the TLX1 shRNA, we wanted to assess whether reintroduction of wild-type TLX1 would reverse the knockdown impact on the expression levels of representative genes selected from the TLX1 signature gene list (see Figure 2B). In the experiments described above, we downregulated endogenous TLX1 using an shRNA (TLX1 shRNA95) directed against the TLX1 coding region. To independently corroborate target gene expression changes and to facilitate rescue experiments by ectopic TLX1 expression, we used a different shRNA (TLX1 shRNA93) directed against the 3' noncoding region of TLX1 mRNA. After lentiviral delivery of TLX1 shRNA93 and sorting for a CD1b^Low^CD55^High ^surface profile, retroviral vectors expressing the coding region of wild-type TLX1 (TLX1 WT) and two mutant TLX1 proteins were stably introduced into the TLX1 knockdown cells (Figure 1D). As shown in Figure 1E, regulation by TLX1 was confirmed for the selected genes (*DTX1*, *GAS1*, *HMGA2*, *L1TD1*, *PLAC8*, *SH3BP5*, *SLC44A1*) (*P *< 0.05, TLX1 WT vs GFP control). *GAS1*, *HMGA2*, *L1TD1 *and *PLAC8 *expression required TLX1 DNA-binding activity since introduction of a DNA binding-deficient form of TLX1 carrying a mutation within the homeodomain (TLX1 N51A) [53] had minimal if any activity (*P *< 0.05, TLX1 WT vs TLX1 N51A).

In other recent work, we reported that TLX1 binds the TLE1 corepressor in an Eh1-dependent manner [49], and we demonstrated that this interaction is important for transcriptional activation of two known TLX1 target genes, *Aldh1a1 *and *Fhl1 *in NIH3T3 cells [54,55]. We therefore wished to determine whether any of the selected TLX1-responsive genes in human T-ALL cells similarly depend on the Eh1 motif. Notably, we found that introduction of a TLE binding-deficient mutant of TLX1 (TLX1 F19E, with a Phe 19 to Glu mutation within the Eh1 motif) [49] significantly diminished the effect obtained by TLX1 reconstitution-of-function for *GAS1*, *PLAC8 *and *SH3BP5 *(*P *< 0.05, TLX1 WT vs TLX1 F19E) (Figure 1E). The data thus suggested that DNA-binding activity and/or TLE interaction are important for TLX1-mediated transcriptional regulation of the studied TLX1 signature genes.

### TLX1 and NOTCH coregulate transcription on a global scale

Toward the identification of transformation-essential TLX1 targets, we employed an elegant approach recently described by Land and colleagues, who found that the gene sets that were coregulated by loss-of-function p53 and Ras activation, but not the targets of either genetic lesion alone, were enriched in genes required for *in vivo *tumorigenic potential [56]. Since activating NOTCH1 mutations are tightly associated with TLX1^+ ^T-ALL [11-13], we were interested in investigating whether there was a potential cooperation between NOTCH and TLX1 in the control of target gene expression underlying leukemic cell growth, especially in view of the role of TLE proteins in NOTCH signaling [41-43]. T-ALL-associated mutations in NOTCH1 occur in the extracellular heterodimerization (HD) domain and/or the C-terminal PEST domain of the protein: HD domain mutations increase the rate of production of the intracellular form of NOTCH1 and mutations that eliminate the PEST domain increase protein half-life [57]. ALL-SIL cells harbor gain-of-function NOTCH1 mutations in both the HD domain and the PEST domain [11]. This NOTCH1 mutant still requires cleavage by γ-secretase to generate the mature intracellular form which translocates to the nucleus to regulate gene transcription. Therefore, we used the γ-secretase inhibitor (GSI) Compound E [11] to downregulate NOTCH pathway signaling in the ALL-SIL cell-derived populations described above expressing three different levels of TLX1. GSI treatment was with 500 nM Compound E and RNA was prepared 24 hours posttreatment as described previously [14]. In total, we generated six different experimental conditions - high, medium or low TLX1 levels treated with GSI or vehicle control (DMSO) - and performed expression profiling experiments using cDNA arrays as described above. Principal Component Analysis indicated that transcription on a global scale was more profoundly affected by downregulation of TLX1 than by inhibition of NOTCH signaling; i.e., larger sets of genes were found to be responsive to changes in TLX1 levels than to GSI treatment (Additional files 1 and 2). Sets of TLX1-responsive genes derived from GSI- or DMSO- treated cells overlapped significantly as did sets of GSI-responsive genes identified under the conditions of high or low TLX1 levels (*P *< 0.0001) (Figure 2A). Using the DAVID gene ontology classification tool, we performed comparative analysis of the functions of the genes responsive to NOTCH in the presence or absence of TLX1 and searched for GO terms uniquely associated with either of these conditions. The best DAVID score for TLX1^+ ^conditions was associated with the GO term "response to external stimulus" (*P *< 0.001, representing 8% of the subset under high TLX1 conditions and *P *< 0.001, representing 6.7% of the subset under medium TLX1 conditions) whereas the best score in the absence of TLX1 expression was associated with the GO term "nervous system development" (*P *< 0.006, representing 8.8% of the subset). This data suggested that expression of TLX1 may influence the functional outcome of NOTCH pathway activation.

Strikingly, the lists of GSI-responsive genes and TLX1-responsive genes overlapped very strongly: more than 60% of the GSI-responsive genes were also controlled by TLX1 (Figure 2A), indicating that a significant fraction of NOTCH target genes in ALL-SIL cells is coregulated by TLX1 (*P *< 0.0001). To quantitatively compare the individual contributions of TLX1 and NOTCH in the regulation of common targets, we identified those genes that are differentially expressed in ALL-SIL cells where TLX1 and NOTCH are "on" versus cells where both factors are "off" (i.e., GSI-treated TLX1^Low ^cells) (Additional file 3). As summarized in Figure 2B, downregulation of TLX1 and inhibition of NOTCH affected the regulation of the vast majority of genes in the same direction. We found that approximately half of the TLX1-induced genes showed the highest expression levels in the presence of NOTCH signaling. Interruption of the transcriptional output of either factor led to the downregulation of these genes. Less than 10% showed a much stronger response to GSI, exhibiting downregulation upon GSI treatment and a somewhat more complex response to TLX1 knockdown. One such example, the NOTCH target gene, *DTX1 *(*Deltex1*) [58], was chosen for validation by qRT-PCR together with 7 TLX1/NOTCH-coinduced genes (*CD1B*, *HMGA2*, *L1TD1*, *OR10R2*, *PLAC8*, *SH3BP5*, *SLC44A1*) and one TLX1/NOTCH-corepressed gene (*GAS1*). The qRT-PCR data reported in Figure 2C is presented as relative expression levels normalized to levels in GSI-treated TLX1^Low ^cells; thus, a positive value indicates positive regulation by TLX1 and activated NOTCH. Regulation by TLX1 in the presence or absence of NOTCH signaling was demonstrated for all of the putative TLX1/NOTCH-coinduced genes examined (*P *< 0.05), and by NOTCH under conditions of high or low TLX1 expression (*P *< 0.05, for at least one TLX1 expression condition; see Additional file 4 for a complete list of *P *values) (Figure 2C). In support of the generality of these latter results, it had previously been reported that *PLAC8*, *SH3BP5 *and *SLC44A1 *were downregulated following NOTCH inhibition with a different GSI in a different T-ALL-derived cell line [59] (see also Additional file 5). Regulation of the NOTCH target gene *DTX1 *by TLX1 was also confirmed in this series of experiments (Figure 2C). As expected, *DTX1 *showed significantly lower levels of expression following GSI treatment (*P *< 0.05). Interestingly, under conditions of NOTCH inhibition, *DTX1 *mRNA levels were higher in TLX1^Low ^cells than in TLX1^High ^cells (*P *< 0.05), reaffirming the complexity of the *DTX1 *response to TLX1/NOTCH coregulation suggested by the microarray data.

We also verified in this series of experiments that *GAS1 *was repressed by TLX1 (*P *< 0.001) (Figure 2C). Since the Eh1 TLE-binding motif mutant of TLX1 (TLX1 F19E) exhibited markedly reduced *GAS1 *repressive activity (*P *< 0.02) (Figure 1E), we decided to directly investigate the potential contribution of TLE corepressors to the *GAS1 *repression mechanism. ALL-SIL cells expressing the TLX1 shRNA95 or pLKO control vector were transduced with lentiviral vectors expressing panTLE shRNAs. As seen in Figure 2D, knockdown of TLE resulted in partial derepression of GAS1 in the presence of TLX1 (*P *< 0.008). In contrast, no effect was observed under conditions of low TLX1 expression (*P *= 0.6), indicating that TLX1 is required for repression of *GAS1*. The magnitude of derepression achieved by TLE knockdown (~3-fold) was much less than that obtained following TLX1 knockdown (~40-fold), presumably due in part to incomplete knockdown of TLE. A representative Western blot shown in Figure 2E indicates that only ~50% downregulation of TLE1 protein levels was obtained (*P *< 0.05). In this series of experiments, GSI treatment also resulted in partial derepression of GAS1 in the presence of TLX1 (~2-fold, *P *< 0.003) (Figure 2D) supporting the data presented in Figure 2C indicating involvement of NOTCH. Notably, knockdown of TLE resulted in partial derepression of *GAS1 *in the presence of activated NOTCH (~3-fold, *P *< 0.008) but had no significant additional effect when NOTCH was inhibited. Collectively, the data for *GAS1 *supported the hypothesis that TLE corepressors can serve as common cofactors in the regulation of certain TLX1/NOTCH-corepressed genes.

To compare the cellular functions regulated by TLX1 and NOTCH together or by either factor separately, we performed a functional classification of gene sets that were enriched for NOTCH only-induced genes, TLX1 only-induced genes and TLX1/NOTCH-coinduced genes. DAVID analysis revealed that all three datasets were enriched for functional GO terms associated with developmental processes. In agreement with the well-recognized survival function of NOTCH in normal and malignant T cells, we found that the gene set induced by NOTCH alone was specifically enriched for GO terms "positive regulation of metabolism" and "apoptosis" [14,15,27], whereas genes only induced by TLX1 were enriched for GO terms associated with "chromatin function and regulation", "proliferation" and "cell-cycle" [60]. Importantly, GO terms associated with "immune system development" and "oncogenesis" only appeared in the set of genes regulated by both TLX1 and NOTCH (Figure 2A). Notably, *HMGA2 *and *PLAC8 *were previously reported to be among the 'cooperation response genes' identified by loss-of-function p53 and Ras activation as contributing to the malignant phenotype in colon cancer [56]. A role of *HMGA2 *in embryonic growth and development has also been described [61-63]. Moreover, the mouse orthologs of 4 of the other 6 TLX1/NOTCH-coinduced signature genes verified by qRT-PCR (*L1TD1*, *PLAC8*, *SH3BP5*, *SLC44A1*) are downregulated during differentiation of mouse embryonic stem cells whereas the TLX1/NOTCH-corepressed target *GAS1 *is activated during embryonic stem cell differentiation (GEO profiles GDS2905 and GDS2906). These observations indicate that the TLX1/NOTCH leukemic signature shares a common component with an embryonic stem cell-like transcriptional program [64]. Furthermore, CD1b, the cell surface marker characteristic of the early cortical thymocyte stage of differentiation arrest associated with the vast majority of TLX1^+ ^T-ALL cases, was also among the targets coregulated by TLX1 and NOTCH.

### TLX1-mediated augmentation of MYC contributes to T-ALL growth regulation

**Figure 3:**
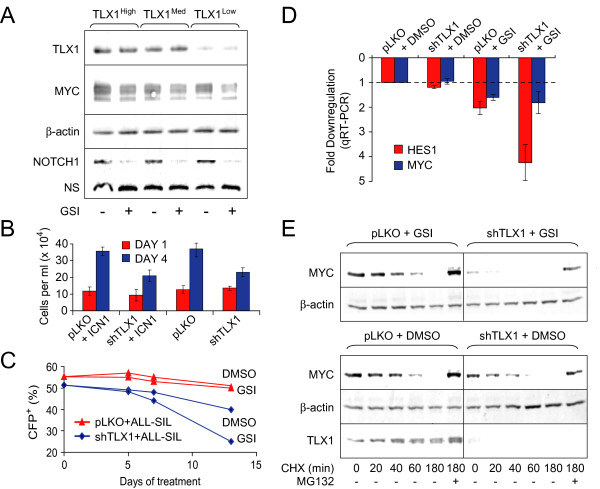
**TLX1 and NOTCH coregulate MYC levels and ALL-SIL growth**. (A) ALL-SIL derivatives expressing the indicated levels of TLX1 were treated for 24 hours with 500 nM Compound E (GSI, +) and 0.05% DMSO (GSI, -) as vehicle control. Whole cell lysates were analyzed for expression of activated NOTCH1 and MYC proteins by Western blotting. A representative blot of 4 biological replicates is shown. (B) Activated NOTCH1 expression does not compensate for the lack of TLX1. ALL-SIL cells expressing the TX1 shRNA95 or pLKO.1-CFP control vector were lentivirally transduced to express a constitutively active form of NOTCH1 (ICN1). Cells were seeded at 1 × 10^5 ^cells per ml in parallel with mock-transduced controls and counted on days 1 and 4 by trypan blue staining. (C) Growth competition experiments in the presence or absence of 500 nM Compound E, indicated as GSI and DMSO, respectively. ALL-SIL cells expressing TLX1 shRNA95 and CFP or pLKO.1-CFP were mixed with parental ALL-SIL cells in equal proportions. Mixed populations were divided, treated with GSI or DMSO control for up to 3 weeks and periodically examined by flow cytometric analysis. (D) TLX1 and NOTCH regulation of *MYC *and *HES1 *mRNA. (E) TLX1 prevents GSI-induced downregulation of MYC protein and extends MYC half-life. Cells were treated with 500 nM Compound E or DMSO control for 24 hours, then assayed for MYC protein levels and stability using 50 μg/ml cycloheximide or 40 μM MG132 treatment for the indicated times.

To understand how TLX1 might modulate NOTCH-dependent transcription, we first examined whether TLX1 influences intracellular levels of NOTCH1. We found that neither TLX1 levels nor sorting for CD1/CD55 affected the levels of activated NOTCH1, whereas, as expected, GSI treatment markedly decreased intracellular levels of NOTCH1 (Figure 3A). We also found that expression of constitutively active form of NOTCH1, ICN1 (encoding the intracellular domain of NOTCH1), does not substitute for the lack of TLX1 in the growth regulation of ALL-SIL cells (Figure 3B). However, as determined by growth competition assay, both factors synergistically regulated ALL-SIL growth, since GSI treatment significantly enhanced the differences between the growth rates of the TLX1-expressing and knockdown populations (Figure 3C). Together, the data suggested that TLX1 and NOTCH utilize different but synergistic mechanisms contributing to the growth of ALL-SIL cells.

We hypothesized that the *MYC *gene might represent the master regulatory hub targeted by both oncogenic lesions because TLX1^+ ^T-ALLs exhibit increased levels of MYC and MYC target genes [33,60] and NOTCH1 directly activates the *MYC *gene at the transcriptional level in T-ALL [14,15]. We therefore investigated whether TLX1 and NOTCH coregulate MYC in ALL-SIL cells. MYC protein levels were found to be lowest in TLX1^Low ^cells treated with GSI (Figure 3A). Notably, TLX1 did not influence *MYC *mRNA levels under any conditions; e.g., *MYC *mRNA levels decreased following GSI treatment regardless of TLX1 expression (*P *< 0.05, GSI- vs DMSO- treated cells) although TLX1 prevented GSI-mediated downregulation of *HES1 *mRNA (*P *< 0.05) (Figure 3D). We asked next if TLX1 expression contributes to the synthesis or stability of the MYC protein. As shown in Figure 3E, TLX1 increased MYC protein levels in the presence of the MG132 protease inhibitor and, in addition, as seen from cycloheximide treatment, prolonged MYC protein half-life. NOTCH also positively contributed to total MYC protein levels, consistent with previously published data showing that NOTCH directly activates *MYC *gene expression in T-ALL [14,15]. The data thus suggested that TLX1 contributes to the NOTCH-MYC oncogenic axis at least in part via augmentation of MYC protein expression and stability.

**Figure 4:**
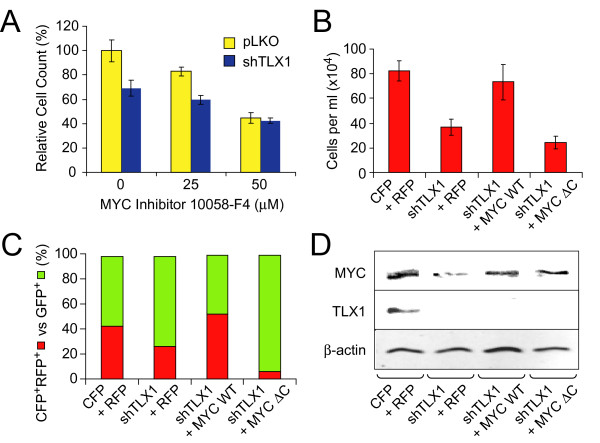
**Role of MYC as a downstream component of the TLX1/NOTCH regulatory network**. (A) Chemical inhibition of MYC (10058-F4 treatment) mimics TLX1 knockdown in ALL-SIL cells expressing shRNA93 versus pLKO.1-CFP expressing TLX1^+ ^controls. (B) Expression of wild-type but not an inactive MYC mutant (MYC ΔC) compensates for the lack of TLX1 expression in ALL-SIL cells treated with GSI. TLX1 shRNA93 was co-expressed with CFP; MYC constructs were coexpressed with RFP. ALL-SIL cells expressing pLKO.1-CFP-TLX1 shRNA93 were transduced with retroviral vectors coexpressing RFP alone, wild-type MYC plus RFP or the MYC ΔC mutant plus RFP and sorted for CFP and RFP expression. Equal numbers were seeded and counted after 2 weeks in culture. CFP + RFP, parental ALL-SIL cells coexpressing CFP and RFP. (C) Growth competition experiments. The same CFP^+^RFP^+ ^ALL-SIL derivatives as in B were mixed with parental ALL-SIL cells expressing GFP in equal proportions. Aliquots were periodically analyzed by flow cytometry; shown are data 2 weeks after initiation of the experiment. (D) Western blot analysis showing MYC protein levels and the corresponding levels of TLX1 for the cell lines studied in B and C.

Next, we assessed the functional role of TLX1-mediated augmentation of MYC and asked how inhibition of MYC activity affects the growth of ALL-SIL cells. ALL-SIL cells were treated with a small-molecule MYC inhibitor, compound 10058-F4 [65,66]. Compound 10058-F4 treatment decreased the growth of ALL-SIL cells to similar levels regardless of TLX1 expression (Figure 4A), suggesting that augmentation of MYC function is a central mechanism of TLX1 contribution to ALL-SIL cell growth. Ectopic expression of wild-type MYC in TLX1^Low ^ALL-SIL derivatives fully compensated for the knockdown of endogenous TLX1. On the other hand, an inactive MYC mutant missing an evolutionarily conserved region called MYC homology box II (MYC ΔC) [67] was not capable of promoting growth of the cells (Figure 4B, C), as expected [68]. Interestingly, we found that a large fraction of TLX1/NOTCH-targeted genes was represented by potential MYC targets when we compared the expression profiling data with MYC CHIP-on-chip data obtained previously for the TLX3^+ ^T-ALL-derived cell line HPB-ALL [58] (Additional file 5). Accordingly, we determined that of 8 representative genes coinduced by TLX1 and NOTCH, 6 were inhibited by 10058-F4 treatment (Table 1). Interestingly, *PLAC8 *expression was upregulated by the MYC inhibitor in agreement with previously published data showing repression of *PLAC8 *in response to MYC overexpression [69]. Since we found that both TLX1 and NOTCH activated *PLAC8*, the data suggested that, at least in this case, there is a more complex interplay between the three transcription factors than simple coactivation.

**Table 1:** TLX1/NOTCH signature gene response to MYC inhibition in ALL-SIL cells

Gene	Fold Downregulation
CD1B	3.6 ± 0.4
HMGA2	2.4 ± 0.3
L1TD1	5.9 ± 0.4
OR10R2	1.9 ± 0.1
PLAC8	0.5 ± 0.1
RAG1	11.1 ± 2.7
SH3BP5	1.2 ± 0.1
SLC44A1	1.5 ± 0.1

Fold downregulation was defined as the ratio of the qRT-PCR values obtained for untreated cells (DMSO) versus cells treated for 2 days with 50 μM of the MYC inhibitor 10058-F4.

### TLX1/NOTCH coregulation of T-cell developmental genes

**Figure 5:**
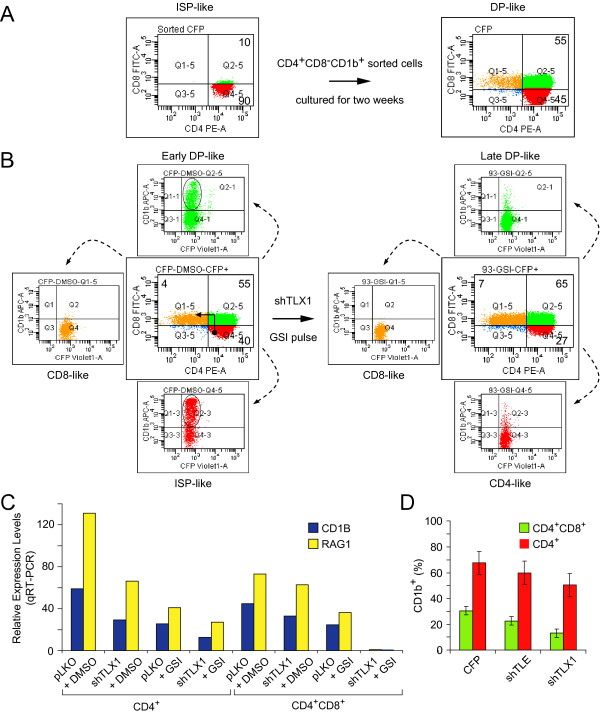
**TLX1 and NOTCH coregulate expression of T-cell developmental genes**. (A) FACS analysis of a CD4^+^CD8^-^CD1b^+ ^ISP-like subpopulation of TLX1^+ ^ALL-SIL cells immediately after sorting (left) and after culture for 2 weeks (right). (B) Transient inhibition of NOTCH and downregulation of TLX1 shifts ALL-SIL populations toward a more differentiated phenotype. Flow cytometry-based comparison of TLX1^+ ^ALL-SIL cells expressing CFP and TLX1 knockdown cells expressing shRNA93 treated by a pulse of GSI. The pulse of GSI consisted of 2 weeks treatment with 500 nM Compound E followed by culture for 1 month. (C) qRT-PCR detection of *RAG1 *and *CD1B *in the same cell lines as in B. The data shown is a representative example for CD4^+^CD8^+ ^(DP) and for CD4^+ ^(ISP-like and SP-like) populations. RNA was extracted from the cells 24 hours after sorting (*P *values for all comparisons are provided in Additional file 4; *n *= 2 biological replicates, each comprising 3 technical replicates). (D) The role of TLE corepressors in TLX1-mediated regulation of CD1b surface expression. Flow cytometric analysis of ALL-SIL expressing shRNA targeting TLX1 or panTLE. The percentage of CD1b^+ ^populations are shown for CD4^+^CD8^+ ^(green) and for CD4^+ ^(red) populations of ALL-SIL cells.

The functional classification of the TLX1/NOTCH-coregulated genes suggested that concerted activity of these oncogenes may alter T-cell development. We previously reported that TLX1 downregulation correlates with a decrease in CD1b surface expression in ALL-SIL cells and in another TLX1^+ ^T-ALL cell line (K3P) [50]. Here we investigated whether inhibition of NOTCH contributes to CD1b surface expression as well. First, we characterized the surface phenotype of ALL-SIL cells in greater detail. Although the majority of the cells (~70%) are CD4^+^CD8^+^CD1b^+/- ^(DP-like), we found that ~30% exhibit a CD4^+^CD8^-^CD1b^+ ^ISP-like phenotype and ~2% exhibit a CD4^-^CD8^+^CD1b^- ^SP-like phenotype. When the CD4^+^CD8^-^CD1b^+ ^ISP-like population was sorted (>90% purity) and returned to continuous culture for 2 weeks, the phenotypic heterogeneity remerged with ~55% of the cells exhibiting a DP-like CD4^+^CD8^+^CD1b^+/- ^phenotype (Figure 5A). Additionally, sorted DP-like populations produced about 2% SP-like cells (data not shown). Thus, the observed heterogeneity of the surface phenotype of the ALL-SIL line resembles a dynamic system of states reminiscent of a hierarchical organization of malignant T-ALL cells [70], with the majority of cells arrested at the early DP stage typical for TLX1^+ ^T-ALL [32-36]. Downregulation of TLX1 by transduction with an shRNA95-expressing vector followed by puromycin selection or an shRNA93- plus CFP-expressing vector followed by FACS caused a marked decrease in CD1b levels in both ISP-like and DP-like populations. In addition, we found that 2 weeks of GSI treatment decreased CD1b surface expression, predominantly affecting the ISP-like populations; however, when TLX1 was knocked down, GSI treatment caused a decrease in the surface expression of CD1b in both DP- and ISP-like populations. Moreover, the effect of GSI treatment was irreversible in the TLX1 knockdown populations since, even when the cells fully recovered 1 month after GSI treatment, CD1b could not be detected on the cell surface. This is illustrated in Figure 5B, where it can be seen that the ALL-SIL population balance was shifted toward more mature phenotypes, with reduced percentages of ISP-like, and increased percentages of DP- and SP-like populations.

To determine whether other developmental genes were regulated in a similar manner, we searched for genes that were coregulated with CD1 family members during normal thymocyte development. *RAG1 *was identified previously as a gene whose expression closely resembles the expression pattern of CD1 [31,32]; specifically, downregulation of both genes occurs at the DP stage, with *RAG1 *downregulation being required for the DP to SP transition [71,72]. We sorted ISP-like and DP-like populations from ALL-SIL cells with or without TLX1 knockdown and transient NOTCH inhibition (2 weeks of GSI treatment or DMSO and 1 month recovery). We examined *CD1B *and *RAG1 *mRNA levels in these populations and observed a striking concordance in their expression changes; e.g., temporary inhibition of NOTCH signaling in TLX1 knockdown populations led to irreversible repression of *CD1B *and *RAG1 *(Figure 5C). In addition, ectopic reexpression of TLX1 in CD1b^- ^cells failed to reactivate these genes (data not shown). Thus, we found that transient downregulation of NOTCH in concert with TLX1 knockdown is required and sufficient to induce irreversible repression of *CD1B *and *RAG1*. Since the silencing of these genes is an important aspect of the normal T-cell differentiation program, the data suggest that TLX1/NOTCH-coregulated maintenance of their expression exemplifies a mechanism underlying the ALL-SIL differentiation arrest. Interestingly, downregulation of TLE caused a significant decrease in the percentage of CD1b^+ ^cells, suggesting that TLX1-TLE interaction is involved in the TLX1-imposed differentiation arrest (Figure 5D).

## Discussion

**Figure 6:**
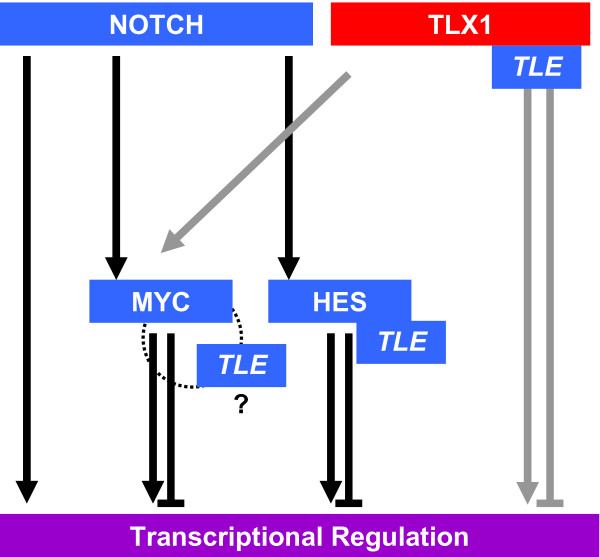
**Schematic summary of regulatory network controlling TLX1/NOTCH signature genes**. NOTCH1 activates transcription of downstream transcriptional regulators MYC and HES. TLX1 augments MYC protein levels. HES represses transcription via interaction with TLE [41-43]. TLX1 interacts with TLE to mediate repression and activation of transcription [49]. MYC binds TLE directly *in vitro *[45]. However, it is not known whether TLE is required for MYC repressor activity (indicated by "?"). We speculate that the genes coregulated by the TLX1/TLE/NOTCH/MYC network are critical for TLX1/NOTCH transforming function in T-ALL.

Several lines of evidence indicate that TLX1 functions as a transcriptional regulator that can either activate or repress gene expression [49,50,53-55,60,73-76]. The situation is complicated by the fact that TLX1 may switch its mode of regulation of the same gene depending on as yet ill-defined tissue-specific factors that may include the availability of transcriptional cofactors, the presence or state of activation of cis-regulatory DNA elements, and/or the expression levels of the TLX1 protein itself (our unpublished observations and [54]). Recently, for two of the best characterized DNA binding-dependent TLX1 targets, *Aldh1a1 *and *Fhl1*, we reported that TLX1 activates transcription via interaction with the transcriptional corepressor TLE [49]. However, the significance of this observation for TLX1-associated leukemogenesis as well as the leukemia-specific downstream targets of TLX1 still remained elusive. In the present work, using a human cell line derived from a TLX1^+ ^T-ALL patient sample we have identified several genes that were upregulated by TLX1 in a DNA binding-dependent manner. Although statistical significance was only reached for a subset of genes examined, an intact Eh1 TLE-binding motif was necessary for maximal effect in all cases with the exception of *L1TD1*. Additionally, we identified a potential direct TLX1 target gene, *GAS1*, which was repressed by TLX1 in a classical TLE and DNA binding-dependent manner [48]. Interestingly, we found that our attempts to overexpress TLE1 by retroviral-mediated gene delivery resulted in massive cell death in TLX1 knockdown derivatives but not in parental ALL-SIL cells (our unpublished observations). Taken together, our data suggest that TLX1 interaction with TLE may be an integral component of TLX1 leukemic function (Figure 6). To explain how TLX1-TLE binding may cause transcriptional activation, we can envisage at least two mechanisms: 1) TLX1-TLE-mediated repression of unknown intermediary transcriptional repressors (referred to as the repressor-of-repressor mechanism [77]); and/or 2) A derepression strategy involving competitive sequestration of TLE from other TLE-dependent transcriptional repressors. To illustrate the validity of the second mechanism, we previously used a well-established HES1-TLE-dependent repressor model involving the *ASCL1*/*HASH1 *gene and showed that ectopic expression of TLX1 dismissed TLE from the *ASCL1 *promoter, preventing HES1-mediated *ASCL1 *repression [49].

The TLE protein family plays a role in a negative loop of regulation of NOTCH as well as facilitating NOTCH repressor function [38,41]. We found that TLX1 coregulated a large proportion of NOTCH-responsive genes. As was confirmed by qRT-PCR, all of the TLX1 targets requiring the Eh1 TLE-binding motif for their full activation were also positively regulated by NOTCH, implicating TLX1-TLE interaction in the derepression of NOTCH-induced genes. Downregulation of TLE levels by pan-TLE targeting shRNA was associated with partial transcriptional derepression of the *GAS1 *gene, supporting the idea that TLE is directly involved in NOTCH- and TLX1-mediated corepression of certain genes. The genes induced by TLX1 in a TLE binding-dependent manner also responded to reduced TLE levels but the effects were variable, consistent with multiple indirect modes of TLE involvement. Also, one of the strongest transcriptional activation responses to TLX1 and NOTCH, exhibited by the *L1TD1 *gene, does not require the Eh1 TLE-binding motif for maximal effect. However, expression of *L1TD1 *and most of the other TLX1-NOTCH signature genes studied were strongly inhibited by the 10058-F4 MYC inhibitor [65,66]. Since we found that TLX1 expression enhances MYC protein levels in ALL-SIL cells and that this is a central facet of TLX1-mediated growth control, we conclude that augmentation of MYC activity is an additional and important aspect of TLX1/NOTCH cooperation. It was previously appreciated that high levels of expression of MYC target genes is a characteristic feature of TLX1^+ ^T-ALLs [33,60]. Thus, our results provide an explanation for these observations and link TLX1 to the NOTCH-MYC signaling axis (Figure 6) [14,58]. In retrospect, since the majority of NOTCH-induced genes in T-ALL are also targeted by MYC [58], it is perhaps not surprising to find that virtually all of the TLX1/NOTCH signature genes that we validated by qRT-PCR negatively responded to inhibition of MYC by compound 10058-F4 treatment. Another level of complexity has arisen with a recent publication showing that mammalian MYC and TLE proteins directly bind *in vitro *[44]. In that study, the investigators also reported that TLE antagonized MYC transcriptional activation of growth-promoting genes in *Drosophila *[44]. It is possible therefore that TLX1-mediated modulation of TLE interactions may derepress MYC target genes as well as NOTCH-responsive genes in T-ALL cells (Figure 6). Finally, promoter analysis of the genes whose expression positively correlated with TLX1 expression showed enrichment for NOTCH-responsive elements (our unpublished observations). Others have shown that TLX1 interacts with SHARP, a corepressor protein directly controlling NOTCH target genes via this element [78,79]. Thus, we speculate that TLX1-SHARP interaction may be an additional point of intersection with the NOTCH signaling pathway.

Since GSI targets all NOTCH receptor family members, we deliberately avoided specifying throughout which NOTCH receptor cooperates with TLX1 in ALL-SIL cells. However, the fact that *NOTCH1 *is mutated in these cells [11], considered together with our Western blot results showing that GSI treatment markedly decreased intracellular levels of NOTCH1, strongly implicates activated NOTCH1 in the observed cooperation with TLX1 [11]. It is important to point out, however, that others have suggested that TLX1 may enhance NOTCH3 signaling in T-ALL cells, although no information regarding protein levels or functional activation of the NOTCH3 receptor was provided [78].

TLX1-activating mutations in T-ALL are associated with an early cortical phenotype, suggesting a direct inhibitory effect preventing the differentiation of thymocytes past this developmental stage [32-36]. In experimental models, we and others have observed that TLX1 expression may lead to differentiation arrest of a broad spectrum of hematopoietic precursors, and that extinction of TLX1 expression released the block allowing continuation of the differentiation program in erythroid precursors conditionally expressing TLX1 [76,80]. Moreover, we recently showed that TLX1 downregulation in ALL-SIL cells correlates with acquisition of a surface phenotype associated with early cortical to late cortical thymocyte development epitomized by downregulation of CD1 family members, partially recapitulating the T-cell differentiation program [50]. Here, we have extended the latter studies to demonstrate that the *CD1B *marker of early cortical thymocytes is coregulated by TLX1 and NOTCH concomitant with maintenance of *RAG1 *expression. Downregulation of *RAG1 *in cortical thymocytes is one of the earliest signs of positive selection during normal thymocyte development [71,72]. In this context, we found that downregulation of TLX1 and NOTCH in ALL-SIL cells leads to irreversible repression of the *CD1B *and *RAG1 *genes, since reexpression of TLX1 in the sorted CD1b^- ^cells or resumption of NOTCH signaling was not sufficient to reactivate their expression. Therefore, the data is consistent with a role of TLX1/NOTCH cooperation in preventing the differentiation-programmed repression of the *CD1B *and *RAG1 *genes rather than one involving transcriptional activation of CD1 and RAG family members. In addition, we found that *CD1B *and *RAG1 *were negatively regulated by MYC inhibition. Collectively, these findings implicate the TLX1/NOTCH-MYC network in maintaining the CD1^+ ^early cortical stage of TLX1^+ ^T-ALL cells.

A major motivation for these studies was to identify potential therapeutic targets that are critical to the malignant phenotype in T-ALL. The study design was modeled after that of Land and colleagues who demonstrated in experimental models of murine and human colon cancer that malignancy was strongly correlated with a class of genes they referred to as 'cooperation response genes' which were synergistically regulated by cooperating oncogenic mutations [56]. Based on their observations that the malignant state depended on a similar set of genes in both experimental systems, the investigators suggested that cooperation response genes may broadly contribute to the generation and maintenance of the cancer cell phenotype in a variety of contexts. It is notable therefore that *HMGA2 *and *PLAC8 *were among the genes that we identified. HMGA2 belongs to the high mobility group AT-hook family of architectural nuclear factors involved in chromatin remodeling and gene transcription. It is predominantly expressed during normal embryonic development, but has been implicated as contributing to the transformed phenotype and poor prognosis of a wide variety of human neoplasms [81,82] including hematologic malignancies [83-85]. The *PLAC8 *gene encodes a cysteine-rich protein of unknown function which has been suggested to be involved in cell survival and transformation [69]. It was the top-ranked cooperation response gene identified by Land and colleagues whose perturbation in both murine and human colon cancer cells inhibited tumor formation in mice [56]. Increased expression of *PLAC8 *has been reported in putative cancer stem cells in liver and breast cancer cell lines [86,87]. More intriguing, the *PLAC8 *gene was among the gene signatures predictive of relapse in ALL patients in two recent studies [88,89]. In particular, in a study of 50 T-ALL patients, *PLAC8 *was one of 5 genes that accurately predicted clinical outcome [89]. The accumulated data thus support the idea that *HMGA2 *and *PLAC8 *may play a central role in the malignant phenotype of a broad spectrum of cancers of diverse origins.

## Conclusions

Despite the fact that the oncogenic function of *TLX1 *and *NOTCH1 *is well established in T-ALL, the mechanistic basis of their cooperation remained to be clarified. Our data suggest that in the process of leukemic transformation TLX1 enhances NOTCH signaling output and that both factors contribute to T-ALL cell survival and differentiation arrest. We believe that a search for the common gates targeted by these cooperating genetic lesions will help to better understand the nature of the disease and lead to the development of more effective and less toxic therapeutic regimens. The TLX1/NOTCH 'cooperation response genes' listed in Table 1, especially *HMGA2 *and *PLAC8*, represent attractive candidates for further studies along these lines.

## Methods

### shRNA knockdown and gene transfer experiments

ALL-SIL cells were cultured as previously described [60]. TLX1 shRNA knockdown analyses were performed as previously reported [50] with the following modifications. TLX1 shRNAs were obtained from the MISSION™ TRC TLX1 shRNA target set (Cat. No. NM_005521, Sigma-Aldrich) cloned into the pLKO.1-puro lentiviral vector backbone [90]: TRCN0000014993 (designated TLX1 shRNA93) targets sequences within the *TLX1 *3' noncoding region and TRCN0000014995 (designated TLX1 shRNA95) targets the *TLX1 *coding region. The pLKO.1-CFP-TLX1 shRNA93, pLKO.1-CFP-TLX1 shRNA95 and pLKO.1-CFP lentiviral vectors were created by replacing the puromycin resistance genes in the corresponding pLKO.1-puro plasmids with the enhanced cyan fluorescent protein (CFP) gene from the MCIN retroviral vector [91,92]. The resulting pLKO.1-CFP lentiviral vector backbone was also used to generate panTLE knockdown ALL-SIL cells. The TLE shRNAs were based on an shRNA (TLE1/4si2) previously shown to efficiently knock down *TLE1 *and *TLE4 *in human leukemia cells [47]. The *TLE *shRNA target sequences were as follows: shRNA1/4, GGTCTGCTTCTCATGCTGCAG, which targets sequences in common to both TLE1 and TLE4; shRNA2, GGTTTGCTTCTCCTGCTGCAG, and shRNA3, AGTCTGCTTCTCCTGCTGCAG, which target the corresponding regions in *TLE2 *and *TLE3*. Double-stranded DNAs including the 21-mer TLE shRNA sequences [90] were ligated into the *Age*I-*Eco*RI sites of pLKO.1-CFP. The TLX1 wild-type, and the TLX1 N51A and TLX1 F19E mutant coding regions were previously described [49,53]; these were inserted into the MSCV-GW retroviral vector that coexpresses the enhanced green fluorescent protein (GFP) gene [93]. *MYC *retroviral expression vectors were constructed by cloning the MYC wild-type and MYC ΔC (amino acids 127-189 deleted) mutant coding regions [67] (provided by Dr. William Tansey, Cold Spring Harbor Laboratory, Cold Spring Harbor, NY) into the MSCV-RW retroviral vector in which the GFP gene in MSCV-GW was replaced with the DsRed-Express2 red fluorescent protein (RFP) gene [94] (provided by Dr. Benjamin Glick, The University of Chicago, Chicago, IL). A lentiviral vector that coexpresses a constitutively active form of *NOTCH1 *encoding the intracellular domain of the human NOTCH1 receptor (ICN1; codons 1770-2555) (provided by Dr. Warren Pear, University of Pennsylvania School of Medicine, Philadelphia, PA) [95] and RFP was previously described [49].

Vesicular stomatitis virus (VSV)-G glycoprotein-pseudotyped lentiviral vector particles were produced by transiently transfecting the lentiviral vector plasmids (15 μg), the packaging plasmid pCMVΔR8.91 (10 μg) and the VSV-G protein envelope plasmid pMD.G (5 μg) into subconfluent human embryonic kidney 293T cells by the calcium phosphate precipitation method [96], and were used to transduce ALL-SIL cells as previously described [50]. Amphotropic retroviral particles were similarly produced by transiently transfecting 293T cells with retroviral vector plasmids (10 μg) and the pCL-Ampho packaging construct (10 μg), and were used to transduce ALL-SIL cells as previously described [60].

### Fluorescence activated cell sorting and analysis

Vector-transduced ALL-SIL cells expressing CFP, GFP and/or RFP were sorted on a FACSAria instrument (BD Biosciences) equipped with 407-nm solid state, 488-nm solid state and 633-nm HeNe lasers [91,92]. Where indicated, the cells were stained with anti-CD1b-Alexa Fluor 647 and anti-CD55-PE monoclonal antibodies prior to sorting. Other monoclonal antibodies included, anti-CD4-FITC, anti-CD4-PE, anti-CD8-FITC and anti-CD8-PE (all purchased from eBioscience). Cell staining was carried out with saturating concentrations of reagents as described [97] and flow cytometry data was analyzed using FACSDiva software (BD Biosciences).

### Cell growth assays

ALL-SIL cell populations expressing fluorescent protein reporters (CFP, GFP or RFP) were mixed in equal proportions and periodically analyzed by flow cytometric monitoring. In some experiments, cell growth was measured using the alamarBlue cell viability and proliferation reagent (Invitrogen) as previously described [50]. Where indicated, ALL-SIL cell populations were treated with the GSI, Compound E, at 500 nM for 24 hours [14] or 2 weeks [11], or with the MYC chemical inhibitor 10058-F4 (both from Calbiochem, EMD Chemicals) at the indicated concentrations as previously described [65,66]. MG132 and cycloheximide were from Sigma-Aldrich. Mock-treated cultures contained 0.05% dimethylsulfoxide (DMSO) as solvent vehicle control.

### Quantitative real-time RT-PCR validation and analysis of genes

Real-time qRT-PCR was performed using the Power SYBR Green reagent (Applied Biosystems) on an ABI Prism 7000 Sequence Detection System as previously described [76,98]. Primers: TLX1 coding (CATCGACCAGATCCTCAACA, CAGCCAAGGCCGTATTCTC); TLX1 3' noncoding (GTCACTGTCCCTCCTGGTGT, GCCTGATCGTAAGGTCCAAA); L1TD1 (ACGCCAGGGTGACTACAAAC, GCTGTCCATCCTTCTGGGTA); OR10R2 (CTTTCTGTGGCCAGGACAAT, AACCCATCACACCCAATAGC); HMGA2 (ACTTCAGCCCAGGGACAAC, CTTCCCCTGGGTCTCTTAGG); DTX1 (GCTAATTGTCTTCGGCCAAC, GCTGGCATCCCTTTAAATCTT); CD1B (GCTCCTTTTGCTATGCCTTG, TATTGCGAATGGGAGAGGAG); RAG1 (TGTTTAATGGCTTCCAAGAGC, ACACAGGTCCCCTGAATCAA); SH3BP5 (GATGCGGTGTTGGTGCTG, AGAAATGGCATCAGGCTCAG); SLC44A1 (TCAAATGCTTGCTATACAATCTGA, CGTAGAACTCTGGATACTCAATGAA); PLAC8 (GGAGAGCCATGCGTACTTTC, CAAGCTGAAGAGGTGTCTGCT); GAS1 (CGGAGCTTGACTTCTTGGAC, CGTCCTGAACACTGCAGCTA). qRT-PCR controls, MAPK1, PGK1 and POLR2A, were selected based on the cDNA hybridization data and confirmed not to show any changes in expression under the experimental conditions studied. Primers: MAPK1 (CCAGATCCTCAGAGGGTTAAAA, ATCACAGGTGGTGTTGAGCA); POLR2A (AAGATCCTTCCTTGCCTGTG, GCTTTGTTCTTCCCGAGGAT) and PGK1 (CAGTTTGGAGCTCCTGGAAG, AGTTGACTTAGGGGCTGTGC). The data were normalized per MAPK1 expression levels.

### Western blotting

Western blotting was performed essentially as previously described [53,60,76,97]. Antibodies were: TLX1(c-18, sc880), MYC (c-33, sc-42 and N-262, sc-764), TLE1(M101, sc-9121) and TLE (C-19, sc-13373) from Santa Cruz; and anti-Cleaved NOTCH1 (Val1744) from Cell Signaling.

### Microarray gene expression analysis

RNA samples were analyzed with a cDNA microarray (TIGR 40 K microarray) as previously described [51]. In brief, total RNA was prepared using Trizol reagent (Invitrogen) and the RNeasy mini kit (QIAGEN) as per the manufacturers' instructions. Expression analysis of ALL-SIL cells for a particular TLX1 level was derived from three to four independent GSI and DMSO control treatments. For statistical analysis, a biological replicate hybridization experiment was defined as an independent treatment. A hybridization experiment consisted of Cy5-labeled cDNA that was reverse transcribed from 15 μg of total RNA and cohybridized with Cy3-labeled cDNA synthesized from an equal amount of the Stratagene Universal Human Reference RNA, as described [99,100]. Hybridizations were performed for 18-24 hours at 42°C followed by washing in decreasing concentrations of SSC at room temperature and spun dry. Microarray platform, image scanning, fluorescence intensity measurements, normalization across replicate experiments, experimental noise determination, cluster analysis and candidate signature gene identification were performed as previously described [101-104].

### Statistical analysis

Unless noted otherwise, the Student's unpaired *t *test was used to compare differences between indicated groups. A *P *value < 0.05 was considered significant.

## Competing interests

The authors declare that they have no competing interests.

## Authors' contributions

IR designed the overall study, performed most of the experiments, analyzed and interpreted data, and wrote the manuscript. TSH performed the FACS and flow cytometric analyses, and edited the manuscript. TVL performed the cDNA microarray experiments. NHL contributed to the design of the cDNA microarray experiments, performed statistical analyses of the microarray data, and edited the manuscript. RGH supervised and contributed to the conception and design of the overall study, analyzed and interpreted data, and edited the manuscript. All the authors read and approved the final version of the paper.

## Supplementary Material

Additional file 1 **TLX1 target genes derived from cDNA platform hybridization experiments of ALL-SIL cells with or without GSI treatment**. TLX1 targets were selected based on correlations of expression levels with TLX1 protein levels (*r *> 0.7 or < -0.7; 1%, 5% or 10% false discovery rate [FDR]; *n *= 3-4). The data is organized in 4 spreadsheets: "PositiveCorrel DMSO control", genes whose expression levels positively correlated with TLX1 protein levels in untreated ALL-SIL cells; "NegativeCorrel DMSO control", genes whose expression levels negatively correlated with TLX1 protein levels in untreated ALL-SIL cells; "PositiveCorrel GSI treated", genes whose expression levels positively correlated with TLX1 protein levels in GSI-treated ALL-SIL cells; "NegativeCorrel GSI treated", genes whose expression levels negatively correlated with TLX1 protein levels in GSI-treated ALL-SIL cells. Click here for file

Additional file 2 **GSI-responsive genes**. The data is organized in 3 spreadsheets: each spreadsheet represents GSI-responsive genes identified under low ("TLDvsTLG"), medium ("TMDvsTMG") or high ("THDvsTHG") levels of TLX1 expression (1%, 5% or 10% FDR; *n *= 3-4). Click here for file

Additional file 3 **Genes differentially expressed under conditions where TLX1 is expressed and NOTCH is activated versus conditions where TLX1 and NOTCH are both inhibited**. The data is organized in 2 spreadsheets: "THDvsTLG", relative expression levels of genes under conditions of high TLX1 levels and treatment with DMSO versus low TLX1 levels and treatment with GSI; "TMDvsTLG", relative expression levels of genes under conditions of medium TLX1 levels and treatment with DMSO versus low TLX1 levels and treatment with GSI. Click here for file

Additional file 4 ***P *values for qRT-PCR analyses of TLX1/NOTCH-coregulated genes studied in Figures **1E, 2C** and **5C. Click here for file

Additional file 5 **ChIP-on-chip significance analysis of MYC, HES1 and/or NOTCH1 promoter occupancy in TLX3^+ ^HPB-ALL T-ALL cells for selected TLX1 target genes**. Data are from Margolin *et al*. (*Proc Natl Acad Sci USA *2009, **106:**244-249). Click here for file
