# Supplementary material for: TLX1 and NOTCH coregulate transcription in T cell acute lymphoblastic leukemia cells
Source: Mol Cancer. 2010 Jul 9;9:181. doi: 10.1186/1476-4598-9-181 (PMC2913983; doi:10.1186/1476-4598-9-181)
Supplement: Additional file 5 — ChIP-on-chip significance analysis of MYC, HES1 and/or NOTCH1 promoter occupancy in TLX3+ HPB-ALL T-ALL cells for selected TLX1 target genes. Data are from Margolin et al. (Proc Natl Acad Sci USA 2009, 106:244-249). [file 1476-4598-9-181-S5.PDF]

ChIP-on-chip significance analysis of MYC, HES1 and/or NOTCH1 promoter occupancy in $TLX3^+$ HPB-ALL T-ALL cells for the TLX1 target genes listed in Figure 1C and for other selected genes described in the text.[1]

Gene[2,3]	MYC		HES1		NOTCH1	
	Rank	FDR	Rank	FDR	Rank	FDR
**TLX1 Targets**						
ABCB10*	2215	1.40E-08	7488		2715	7.58E-02
ATF1	985	3.86E-11	7839		6033	
CD1B	3519	8.47E-07	715	2.23E-04	5674	
CD59	5225		9823		14541	
CSMD1	-		-		-	
DDX3Y	2196	1.27E-08	8985		11875	
DNAJC12	1505	6.55E-10	555	4.77E-05	13045	
FHL1	1898	3.87E-09	2162	1.83E-02	5823	
GAS1*	4535	1.22E-05	3028	4.87E-02	4151	
HMGA2*	9675		2138	1.75E-02	12894	
IRS1	1059	5.52E-11	4990		7575	
ITPR2	2757	8.82E-08	4045		3665	
L1TD1 / ECAT11	13128		9490		14902	
MAF	7093		554	4.73E-05	768	5.10E-05
OGN	4490	1.12E-05	3322		4920	
PCAF	5586		3906		11894	
PLAC8*	9949		10238		3230	
PLCL1	16054		2911	4.38E-02	16404	
SH3BP5*	7783		11861		9499	
SLC44A1 / CDW92*	6291		3163	5.46E-02	3568	
SLFN5 / MGC19764	12159		6401		9586	
TOB1	6745		15500		14973	
UTY	5859		8249		5533	
CCR7[†]	7739		3310		5047	
CD55 / DAF	1276	2.01E-10	388	5.44E-06	740	3.76E-05
RAB3GAP1	6627		3475		6557	
RAG1[†]	4845	2.57E-05	7618		1362	2.02E-03
RHOU	9536		15629		12240	
**NOTCH1 Targets**						
DTX1	4344	7.88E-06	15480		4768	
HES1	1060	5.52E-11	128	1.14E-08	588	1.06E-05
MYC	1360	2.96E-10	370	4.30E-06	895	1.36E-04

[1]HPB-ALL T-ALL cells contain a t(5;14)(q35;q32.2) translocation that activates the *TLX3* gene (a paralog of *TLX1*) on chromosome 5 by juxtaposition with T cell regulatory elements downstream of the *BCL11B* gene on chromosome 14 (MacLeod *et al*., Genes Chromosomes Cancer 37:84-91, 2003). The gene expression profiles of $TLX3^+$ T-ALL cells are closely related to those of $TLX1^+$ T-ALL cells (Ferrando *et al*., Cancer Cell 1:75-87, 2002; Soulier *et al*., Blood 106:274-286, 2005). The surface phenotypes of most $TLX1^+$ T-ALL cells are $CD1^+$, $CD4^+$, $CD8^+$ and $CD3^-$ (resembling early cortical thymocytes) while those of $TLX3^+$ T-ALL cells are $CD1^{+/-}$, $CD4^+$, $CD8^+$ and $CD3^+$ (resembling early cortical thymocytes with acquired CD3 surface expression); ALL-SIL cells are $CD1b^+$, $CD4^+$, $CD8^+$ and $CD3^-$ while HPB-ALL cells are $CD1b^+$, $CD4^+$, $CD8^{+/-}$ and $CD3^+$.

Data are from Margolin *et al*. (PNAS 106:244-249, 2009); ChIP-on-chip ($ChIP^2$) assays were performed in HPB-ALL cells using Agilent Human Proximal Promoter Microarrays (containing 16,697 promoters). The platform contains ~4-5 probes per gene covering -0.8 kb to +0.2 kb (relative to the transcription initiation site) of human transcripts from the UCSC hg17/NCBI release 35 (May 2004).

Promoters were ranked according to a corrected *P* value for each gene (P_k) representing the probability that the most significant region on the gene's promoter is bound by the transcription factor indicated (see the table below for the *P* values and location of the most significant 500-bp region bound by each transcription factor on each of the promoters). A false discovery rate (FDR) was calculated for each gene *k* according to the formula, $FDR_k = G \times P_k/r_k$, where G represents the total number of genes on the array (16,697), P_k represents the corrected *P* value for each gene *k*, and r_k represents the rank of gene *k*. The FDR thus represents the percentage of genes with ranks lower than the current gene that are expected not to be bound by the transcription factor. The lowest-ranking validated gene before a false-positive was encountered was 4,901 for MYC, 3,247 for HES1 and 2,958 for NOTCH1.

[2]FDR values are shown for *italicized* TLX1 target genes which have predicted binding sites for MYC, HES1 and/or NOTCH1 based on their $ChIP^2$ ranking (i.e., within the top 4,901, 3,247 and 2,958 ranked genes for MYC, HES1 and NOTCH1, respectively).

[3]Genes highlighted in **blue boldface type** are regulated by NOTCH1 in ALL-SIL cells (see Figure 2).

*The ABCB10 promoter (-0.7 kb to +0.3 kb relative to the transcription initiation site) was identified as one of 876 promoters bound by MYC in Daudi human Burkett's lymphoma cells (Li *et al*., Proc. Natl. Acad. Sci. USA 100:8164-8169, 2003);

GAS1 is regulated by constitutive MYC expression in Rat1a rat embryo fibroblasts (Lee *et al*., Proc. Natl. Acad. Sci. USA 94:12886-12891, 1997) and in IMR90 human fetal lung fibroblasts (O'Hagan *et al*., Nat. Genet. 24:113-119, 2000);

HMGA2 is indirectly regulated by MYC through repression of let-7 microRNAs (Chang *et al*., Proc. Natl. Acad. Sci. USA 106: 3384-3389, 2009; Mayr *et al*., Science 315:1576-1579, 2007; Lee and Dutta, Genes Dev. 21:1025-1030, 2007; Shell *et al*., Proc. Natl. Acad. Sci. USA 104:11400-11405, 2007);

PLAC8 is regulated by constitutive MYC expression in murine IL-3-dependent 32D myeloid cells (Rogulski *et al*., Oncogene 24:7524-7541, 2005).

MYC stimulates nuclearly encoded mitochondrial genes and mitochondrial biogenesis (Li *et al*., Mol. Cell. Biol. 25:6225-6234, 2005; Dang *et al*., Clin. Cancer Res. 15:6479-6483, 2009). In addition to ABCB10 (Chen *et al*., Proc. Natl. Acad. Sci. USA 106:16263-16268, 2009), SH3BP5 Wiltshire *et al*., Biochem. Soc. Trans. 32:1075-1077, 2004) and SLC44A1 (Michel and Bakovic, FASEB J. 23:2749-2758, 2009) are mitochondrial proteins.

†CCR7 (not included on the TIGR 40K microarray) is regulated by TLX1 (Riz *et al*., Br. J. Haematol. 145:140-143, 2009) and by NOTCH1 through NF-κB (Vilimas *et al*., Nat. Med. 13:70-77, 2007; Buonamici *et al*., Nature 459: 1000-1004, 2009);

RAG1 was identified as a NOTCH1 target in human MOLT4 (Dohda *et al*., Exp. Cell Res. 313:3141-3152, 2007) and murine T6E (Weng *et al*., Genes Dev. 20:2096-2109, 2004) T-ALL cells.

Locations and *P* values of the most significant 500-bp region on each promoter bound by MYC, HES1 and/or NOTCH1 in TLX3^{+} HPB-ALL T-ALL cells for the TLX1 and NOTCH1 target genes listed above.

Gene	MYC		HES1		NOTCH1	
	Probe location	Probe *P* value	Probe location	Probe *P* value	Probe location	Probe *P* value
ABCB10	226001574	1.574693E-06	226001574	4.650879E-02	226001574	4.611823E-04
ATF1	49444076	3.369876E-08	49443811	6.996551E-02	49444076	1.091585E-01
CD1B	155114012	4.827880E-05	155114012	2.671249E-06	155114163	7.147826E-02
CD59	33714312	2.259602E-06	33701532	6.333860E-02	33714770	1.409037E-01
CSMD1	Not Included	-	Not Included	-	Not Included	-
DDX3Y	13455242	5.490275E-09	13453768	5.897151E-02	13454955	1.449824E-01
DNAJC12	69267922	3.583383E-07	69268054	2.108611E-05	69268891	2.503715E-01
FHL1	134954804	1.534947E-07	134954804	5.251865E-04	134954529	1.277807E-01
GAS1	86792001	1.262689E-04	86792576	2.380969E-03	86792333	5.190079E-02
HMGA2	64504055	5.366990E-02	64503671	5.612401E-04	64504055	5.185487E-01
IRS1	227488710	1.317876E-07	227489418	6.083555E-03	227490497	1.099950E-01
ITPR2	26877817	3.129174E-05	26877285	5.011285E-03	26877817	9.443042E-03
L1TD1 / ECAT11	62372322	9.290709E-01	62372322	3.503655E-02	62372562	1.949010E-01
MAF	78192155	7.369343E-03	78192270	6.125894E-05	78192270	2.370280E-06
OGN	92246366	2.950516E-05	92246366	2.674132E-03	92246366	1.612727E-02
PCAF	20055823	3.844639E-04	20056131	5.494025E-03	20057266	1.548226E-01
PLAC8	84392974	1.184440E-02	84393454	1.901696E-01	84393454	1.942584E-02
PLCL1	198773635	3.518747E-01	198773635	1.849147E-03	198773635	2.633496E-01
SH3BP5	15348629	3.607671E-03	15349643	2.642355E-01	15349643	1.724186E-01
SLC44A1 / CDW92	105086032	1.252660E-03	105085753	1.484255E-02	105085753	1.406197E-02
SLFN5 / MGC19764	30593562	2.345762E-01	30593562	3.224333E-02	30594063	4.127735E-01
TOB1	46296713	6.320553E-03	46296713	1.879201E-01	46297099	1.540183E-01
UTY	14029820	5.165812E-04	14030191	3.736595E-02	14030049	6.188269E-02
CCR7	35975389	6.527389E-03	35975389	6.042224E-03	35975045	1.178153E-01
CD55 / DAF	203883407	2.402585E-09	203883139	1.346520E-05	203883407	4.870725E-06
RAB3GAP1	135643651	1.157059E-03	135643484	1.229370E-02	135643484	1.361635E-01
RAG1	36545988	4.703435E-05	36546303	1.122017E-01	36545988	8.250342E-05
RHOU	225177473	6.235436E-02	225177287	1.340582E-01	225177287	3.910963E-01
DTX1	111958770	2.630991E-05	111957888	4.181230E-01	111958582	3.221684E-02
HES1	195335670	2.555402E-09	195336561	2.402585E-09	195336561	4.948269E-08
MYC	128817655	6.742223E-07	128817439	4.273011E-06	128816561	2.659592E-05
